# Optimal Recovery of Valuable Biomaterials, Chondroitin Sulfate and Bioapatites, from Central Skeleton Wastes of Blue Shark

**DOI:** 10.3390/polym12112613

**Published:** 2020-11-06

**Authors:** José Antonio Vázquez, Javier Fraguas, Pío González, Julia Serra, Jesus Valcarcel

**Affiliations:** 1 Group of Recycling and Valorisation of Waste Materials (REVAL), Marine Research Institute (IIM-CSIC), Eduardo Cabello, 6. Vigo, 36208 Galicia, Spain; xavi@iim.csic.es; 2 New Materials Group, Department of Applied Physics, Campus Lagoas-Marcosende, University of Vigo, IISGS, MTI, 36310 Vigo, Spain; pglez@uvigo.es (P.G.); jserra@uvigo.es (J.S.)

**Keywords:** chondroitin sulfate isolation, *Prionace glauca* skeletons, bioapatite recovery, physicochemical characterization of biomaterials, process optimisation

## Abstract

The industrial filleting of blue shark (*Prionace glauca*) led to the generation of a large number of central skeletons of low interest to fishmeal plants handling such wastes. In this context, the present study describes the optimization of the hydrolysis process (pH 8.35, T 58 °C, 1% (*v/w*) of alcalase and t = 4 h) to produce chondroitin sulfate (CS) together with the recovery of bioapatites. Then, that hydrolysate was chemically treated with an optimal alkaline-hydroalcoholic-saline solution (0.48 M of NaOH, 1.07 volumes of EtOH and 2.5 g/L of NaCl) and finally purified by ultrafiltration-diafiltration (30 kDa) to obtain glycosaminoglycan with a purity of 97% and a productive yield of 2.8% (*w*/*w* of skeleton). The size of the biopolymer (CS) was of 58 kDa with prevalence of 6S-GalNAc sulfation (4S/6S ratio of 0.25), 12% of GlcA 2S-GalNAc 6S and 6% of non-sulfated disaccharides. Crude bioapatites were purified by pyrolysis and FT-Raman and XRD techniques confirm the presence of hydroxyapatite [Ca_5_(PO_4_)_3_(OH)], with a molar mass of 502.3 g/mol, embedded in the organic matrix of the skeleton. The mineralized tissues of blue shark are promising marine sources for the extraction of high value biomaterials with clinical application in bone and tissue regeneration and are still completely unexplored.

## 1. Introduction

Thousands of tons of fish by-products are generated around the world in the filleting of commercial fish species for the production of marine foods. In many cases those wastes are managed as solid urban fraction and in other occasions they are substrates for the production of fish meal [1,2], although many of them are valuable sources of biomaterials that can be used in the formulation of pharmacological, nutraceutical and biomedical devices [3,4,5]. The production of fish meal has been a well-established and very profitable business for several decades, in which the final benefits are directly proportional to the content of protein. This is the main reason for which substrates derived from the central skeletons, bones and heads of fish are not welcome in meal plants, since the fish meal obtained has a low concentration of protein (<60%) and high level of ash (>25%).

In the industrial processing of blue shark (*Prionace glauca*), after muscle separation, central skeletons are disposed in the plants as residues. This by-product supposes around 10–20% of the total weight of the shark and they are formed of vertebral discs and intervertebral fibrocartilage [6] rich in minerals type-apatite, proteins and glycosaminoglycans (GAGs). The valorization of shark skeleton, still unexplored, is an important step for the sustainability of shark fisheries and may lead to the joint recovery of the apatites and GAGs fraction of great relevance in different pharma applications [7,8,9].

GAGs are a family of linear polymers composed of repeating O-linked disaccharide units that are included in the cell surface and in the extracellular matrix of most animal tissues. Because of its habitual association with proteins, they maintain the structure of certain tissues (such as, for example, the cartilage) and are also involved in the proliferation, differentiation, migration and communication of cells [10]. As a consequence of these biological activities, the number of applications of GAGs in the field of tissue regeneration is increasing and, concomitantly, new sources of these biopolymers with varied chemical properties have been recently studied [11,12,13]. Among GAGs, chondroitin sulfate (CS) is one of the most abundant and consists of the repetition of glucuronic acid (GlcA) and N-acetyl galactosamine (GalNAc) linked by alternating β-(1→ 4) and β-(1→3) glycosidic bonds, which can be sulfated at different positions of both monosaccharides. Marine substrates are valuable sources of CS that have reported multiple biological and structural properties [14,15,16], which are dependent on the sulfation pattern and molecular weight of the biopolymer [17,18,19]. Both issues are also influenced by the origin of the substrate (marine species and kind of tissue) and the processes applied for its purification. In this context, the use of sustainable steps for its isolation is fundamental to follow the principles of the circular economy [20,21,22]. Mathematical tools, such as response surface methodology, are integral to optimize the different processes needed to maximize the recovery of CS minimizing the time, reagent and energy demand [23,24,25].

Apatites are bioactive bioceramics which belong to the family of calcium phosphates and, in particular, hydroxyapatite (HA), Ca_10_(PO_4_)_6_(OH)_2_. This basic composition enriched by groups and elements in trace concentrations, as CO_3_^2^^−^, HPO_4_^2^^−^, Na^+^, Mg^2+^, Sr^2+^, K^+^, Cl^−^ and F^−^, constitute the mineral fraction of mineralized tissues, as teeth, skeleton and bones. As a bioactive material, HA has the ability to induce its biological integration in living tissues, and, therefore, to promote the activity of bone tissue forming cells, and activate the adhesion and proliferation of osteoblasts. HA-based materials have attracted great interest in clinical applications as bone grafts, being those of biological origin (mainly bovine, porcine and marine) which is preferred to synthetic biomaterials, given that their behaviour is biologically more active due to the role of the essential elements in the bone defect healing. In previous works, new marine biological sources of apatites are being investigated [7,8]. The potential of the discards from the fishing industry is being explored and, in particular, the extraction of bioapatites from distinct mineralized tissues, shark jaws and teeth, as promising biomaterials for bone regeneration [7,8]. With this goal, the characterization of blue shark vertebrae to identify potential HA as valorization products is of great interest.

The aim of this study was to maximize the processes for the joint recovery of CS and bioapatites from the central skeleton of the blue shark, a substrate completely unexplored. The main variables that affect the enzyme hydrolysis of the skeleton, the selective precipitation and membrane purification of CS, were mathematically modelled and optimized. Finally, chemical features and composition of CS and bioapatite were extensively determined by gel permeation chromatography (GPC), NMR, SAX-HPLC, Raman spectroscopy and X-ray diffraction.

## 2. Materials and Methods 

### 2.1. Wastes from Blue Shark Processing

**Figure 1:**
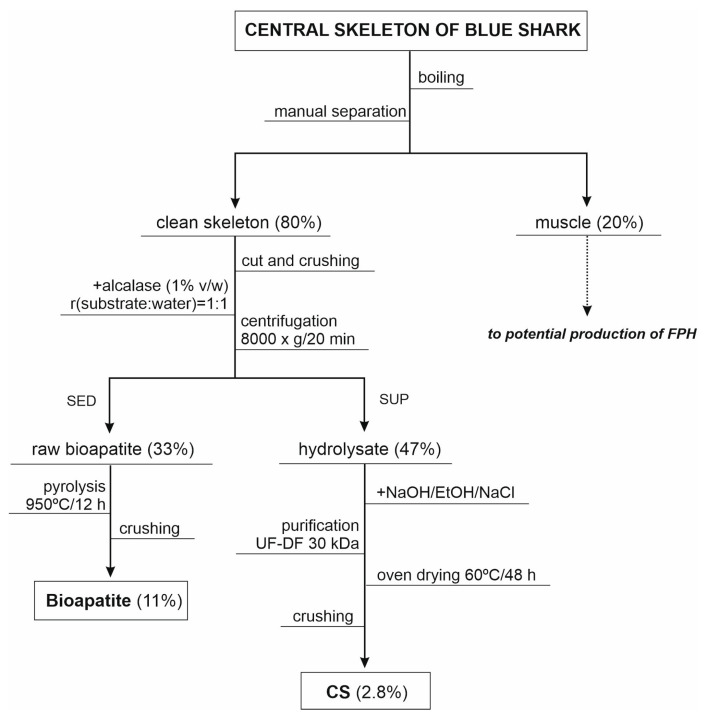
Flowchart of steps studied for the isolation of chondroitin sulfate (CS) and bioapatite from skeleton wastes of blue shark. Percentages of products recovery (*w*/*w* of initial skeleton) are shown in brackets. FPH: fish protein hydrolysates, SED: sediment and SUP: supernatant.

Central skeletons of the blue shark (*Prionace glauca*) were kindly supplied by Protea Productos del Mar S.A. (Marín, Spain). They were generated as wastes after fillet preparation and directly stored at −20 °C. These materials were firstly boiled in water at 90 °C for 20 min and then manually cleaned for the separation of muscle remains (Figure 1). The clean skeletons were cut in a bandsaw for meat and bones, then crushed in a meat grinder (obtained pieces around 0.25–1.0 cm), and stored at −20 °C until use. Although the rest of muscle separated was not a motive of study in this manuscript, it could be used as substrate for the production of fish protein hydrolysates (FPH) mediated by an endogenous protease as, for example, alcalase or papain under controlled conditions.

### 2.2. Optimization Experiments

Two experimental designs were sequentially executed to optimize: (a) the joint influence of temperature (*T*) and pH on the skeleton hydrolysis by alcalase; (b) the combined effect of ethanol volume (*E*) and NaOH concentration (*N*) on the recovery of chondroitin sulfate (CS), and corresponding purity, from skeleton hydrolysates after chemical selective precipitation. In both cases, rotatable second order designs were performed [26] according to the conditions defined in Table 1 (that is, 13 experiments combining the different levels of the independent variables, including 5 replicates in the center of the experimental domain). The estimation of the empirical Equation defining the relationship between the independent variables (*T* and *pH* or *N* and *E*) and the responses analyzed (*Y*) was calculated by orthogonal least-squares method, simultaneously with the multivariable statistical analysis of the results [27]. The theoretical form of the polynomial Equations generated are:

(1) $$Y=b_{0}+b_{1}T+b_{2}pH+b_{12}TpH+b_{11}T^{2}+b_{22}pH^{2}$$

(2) $$Y=b_{0}+b_{1}N+b_{2}E+b_{12}NE+b_{11}N^{2}+b_{22}E^{2}$$

where *Y* is the dependent variable evaluated, b_0_ is the intercept, b_1_ and b_2_ are the parameters for the linear effects, b_12_ is the term of the joint effect of the independent variables, b_11_ and b_22_ are the parameters of the quadratic effects. For the statistical analysis of the results, the significance of the coefficients was evaluated by Student’s *t*-test (*α* = 0.05). The coefficients of determination and adjusted coefficients of determination (*R^2^* and $R_{adj}^{2}$) were calculated to establish goodness-of-fit, and the two mean squares ratios from the Fisher *F*-test (*α* = 0.05) were determined to confirm the consistency of the polynomial models: *F*1 = model/total error, the equation is acceptable when *F*1 ≥ $F_{\mathrm{den}}^{\mathrm{num}}$; and *F*2 = (Model + lack of fitting)/model, the equation is acceptable when *F*2 ≤ $F_{\mathrm{den}}^{\mathrm{num}}$. $F_{\mathrm{den}}^{\mathrm{num}}$ are the theoretical values for *α* = 0.05 with corresponding degrees of freedom for the numerator (num) and denominator (den).

In the experiments of hydrolysis, shark skeletons were treated with a serine-type endoprotease alcalase 2.4 L (Novozyme Nordisk, Bagsvaerd, Denmark) at enzyme/substrate ratio of 24 U/kg (1% *v*/*w* of fresh skeleton) for 4 h, employing a mixture (solid:water) of 1:1, in each of T/pH conditions summarized in Table 1, and maintaining continuous agitation of 200 rpm. These runs were carried out in a 100 mL-pH-stat-reactor (Metrohm 902 Titrando, Metrohm Hispania, Madrid, Spain) equipped with temperature and agitation control. At the end of hydrolysis, samples were centrifuged at 8000× *g* for 20 min and the supernatants analyzed as described in Section 2.4.

In the studies of the selective precipitation of CS, NaOH and EtOH were added to the enzyme hydrolysates at the levels showed in Table 1, plus 2.5 g/L of NaCl in all cases. These mixtures were continuously agitated at 100 rpm, under room temperature, for 2 h; subsequently sediments generated after centrifugation (8000× *g* for 20 min) were re-suspended in water, neutralized using 6 M HCl and analyzed by protocols defined in Section 2.4. Based on the optimal conditions defined by both factorial designs (see results and discussion section), a larger amount of skeletons was processed in order to: (1) confirm the validity of these experimental conditions and (2) to produce an enough volume of impure CS solution for its final purification by membrane technology. For this scale-up of the hydrolysis process, a 5 L pH-stat was utilized similar to those defined in a previous report [23].

### 2.3. Membrane Purification of CS 

For increasing the purity of the CS solutions obtained after chemical treatment, an ultrafiltration membrane (UF) with a 30 kDa cut-off weight (spiral polyethersulfone, 0.56 m^2^, Prep/Scale-TFF, Millipore Corporation, Burlington, MA, USA) was used. The process was performed in a regimen of full recirculation of retentate in two stages: (1) initially UF mode was applied for the concentration of CS solution until a volumetric factor of concentration of 4 and maintaining a constant pressure of 1 bar at a flow of 250–300 mL/min, and then (2) dialfiltration mode (DF) at a constant volume (filtration flow = water intake flow) was chosen for the elimination of protein and salts [28] performing at least 7–8 diavolumes. Samples from UF and DF steps were collected at different concentration factors and diavolumes for protein and CS analysis. DF experimental trends were mathematically modeled using an integrated first-order function, exponential Equation (3), [29]:

(3) $$R=R_{f}+R_{0}e^{-\left(1-s\right)D}$$

where *R* is the concentration of permeable CS or soluble protein in the retentate (% from the level at initial DF), *R_f_* the retentate concentration of CS or protein in the asymptotic phase (%), *R_0_* the permeate concentration (%), *D* the relative diavolume (volume of added water/constant retentate volume), and *s* is the specific retention of CS or protein ranging from 0 (the solute is totally filtered) to 1 (the solute is completely retained). At the end of diafiltration, pure CS solutions were dried in an oven at 60 °C for 2 days.

### 2.4. Chemical Determinations and Biopolymer Characterization

The content of total soluble proteins (Pr) was measured in all samples by the Lowry method [30] and the presence of CS by hydroxydiphenyl protocol [31,32]. In addition, the purity in CS (*I_p_*) regarding the protein content, calculated as *I_p_* (%) = CS × 100/(CS + Pr), was also determined in all experiments.

Absolute molecular weight of CS was determined by GPC (gel permeation chromatography) with light scattering detection as previously described [33]. Briefly, an Agilent 1260 HPLC equipped with a pump (G1311B), automatic injector (G1329B), column oven (G1316A), refractive index detector (G1362A) and dual-angle static light scattering detector (G7800A) was used for chromatographic separation of CS on a set of Suprema columns (PSS, Germany): precolumn (5 µm, 8 × 50 mm), 30Å (5 µm, 8 × 300 mm), 100Å (5 µm, 8 × 300 mm) and ultrahigh (10 µm, 8 × 300 mm). Temperature was set to 30 °C in the column oven and light scattering detector and to 40 °C in the refractive index detector. The mobile phase consisted of 0.1 M NaN_3_ and 0.01 M NaH_2_PO_4_ at pH 6.6. PEO (polyethylene oxide) standard and CS samples were dissolved in the mobile phase at 1 g/L and stirred overnight in an orbital shaker at 200 rpm. A volume of 100 µL of standard and sample was injected onto the system and eluted at 1 mL/min. A PEO standard of Mw 106 kDa and PDI (polydispersity index) 1.05 from PSS (Mainz, Germany) was used to determine the instrumental constants of both detectors. Absolute molecular weight estimations were made based on refractive index increments (dn/dc) calculated from the signal of the refractive index detector.

Disaccharide composition and purity of samples were studied by NMR and SAX-HPLC (strong anion exchange chromatography) as previously described [33]. NMR spectra were recorded at 600 MHz on a Bruker DPX 600 cooled down to 10 °C, and analyzed with MestReNova 10.0.2 (Mestrelab Research, Santiago de Compostela, Spain). CS samples were dissolved in D_2_O at 40 g/L. For SAX-HPLC analysis CS samples were depolymerized with chondroitinase ABC (Proteus vulgaris, EC 4.2.2.4., Sigma-Aldrich, Prod. No. C2905, St. Louis, MO, USA.) at 0.2 U enzyme mg^−1^ of CS, pH 8 (0.05 M Tris-HCl:0.15 M NaAc), 37 °C, for 24 h. At the end of the reaction the enzyme was inactivated (70 °C, 25 min), the samples were then centrifuged (12,857 g, 30 min) and filtered through 0.2 µm polyethersulfone (PES) syringe filters. Quantification was made by external calibration with unsaturated disaccharide standards (Grampenz, UK): GlcA-GalNAc 0S; GlcA-GalNAc 4S; GlcA-GalNAc 6S; GlcA 2S-GalNAc 6S; GlcA-GalNAc 4,6S; and GlcA 2S-GalNAc 4S. The percentage of each disaccharide unit was calculated as the mass proportion relative to the sum of all CS units quantified. Chromatographic analysis was carried out in an Agilent 1200 HPLC system (Agilent, Santa Clara, CA, USA) equipped with binary pump (G1312A), column oven (G1316A) and UV-vis detector set to 232 nm (G1314B). A volume of 20 µl of standards and samples was injected into the system and separated in a Waters Spherisorb SAX column (5 µm, 4.6 × 250 mm) at 1.5 mL min^−1^. Gradient elution was performed with 50 mM NaCl pH 4 (A) and 1.2 M NaCl pH 4 (B) under the following program: From 0 to 5 min 100% A; from 5 to 25 min a linear gradient starting with 100% A and ending with 76% A.

Bioapatite was obtained by pyrolysis of crude bioapatite at 950 °C for 12 h with heating ramp of 2 °C/min and cooling ramp of 20 °C/min to remove the organic matter (Figure 1). Physicochemical characterization of crude bioapatite was performed by FT-Raman spectroscopy for evaluation of the functional groups and X-Ray Diffraction (XRD) for structural measurements. Raman spectra were collected in the range of 250 to 4000 cm^−1^ at a resolution of 4 cm^−1^ and 64 scans, using a FT-Raman Bruker RFS100 (Bruker, Billerica, MA, USA) equipped with a Nd: neodymium-doped yttrium aluminum garnet (YAG) laser of 1 W emitting 1064 nm radiation. XRD analysis was performed using a Philips diffractometer, fitted with a PW1710 control unit, a PW1820/00 goniometer and an Enraf Nonius FR590 generator operating at 40 kV and 30 mA. The diffraction patterns were obtained for 10° < 2θ < 60° with a step size of 0.02°.

### 2.5. Numerical and Statistical Evaluations

Response surfaces from second-order factorial designs and statistical analysis were calculated in an Excel-spreadsheet (Microsoft Corporation, Redmond, WA, USA) constructed for this type of evaluation. Numerical and statistical results were moreover confirmed by Statistica v. 8.0 software (StatSoft, Inc, Tulsa, OK, USA). Data fitting of diafiltration-ultrafiltration (UF-DF) data were performed by minimisation of the sum of quadratic differences between observed and model-predicted values, using the non-linear least-squares (quasi-Newton) method provided by the macro ‘Solver’ of the Excel-spreadsheet. 

## 3. Results and Discussion

### 3.1. Production of CS from Blue Shark Skeletons

**Figure 2:**
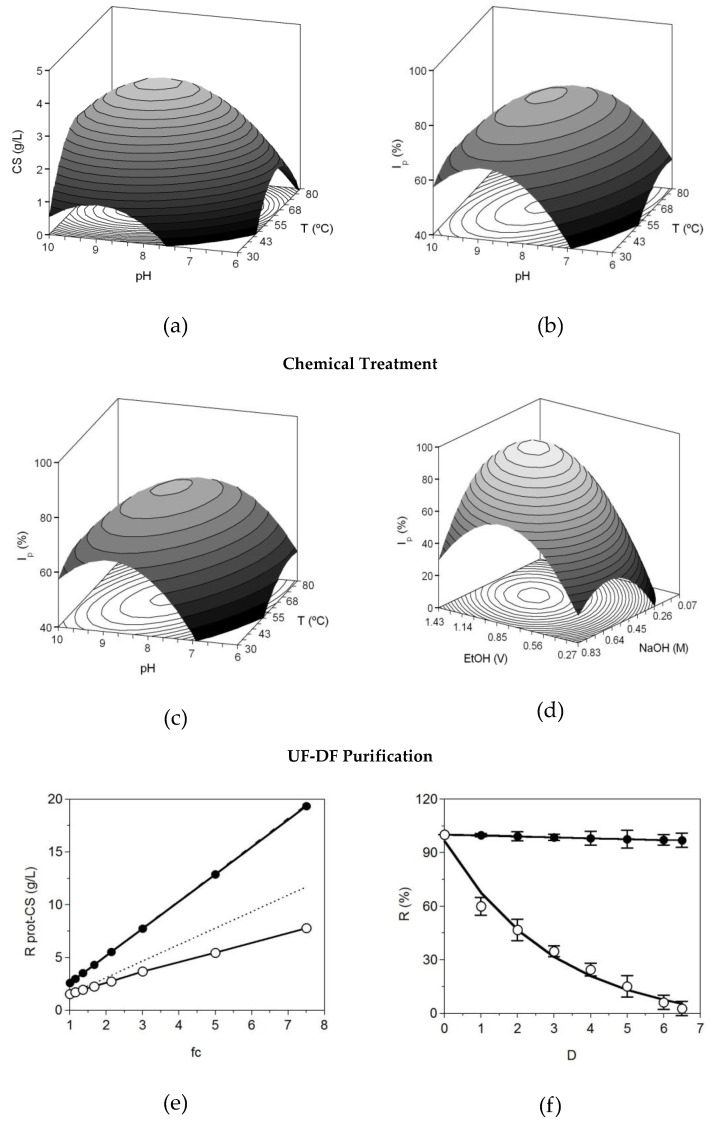
**Top**: Theoretical surfaces obtained from the equations shown in Table 1 defining the combined effect of pH and T on (**a**) chondroitin sulfate concentration (CS) and (**b**) CS purity (*I_p_*). **Middle**: theoretical surfaces also described by equations from Table 1 for the effect of NaOH and ethanol concentration on CS (**c**) and *I_p_* (**d**). **Bottom**: purification of CS samples by UF-DF at 30 kDa; (**e**), concentration of retained protein (○) and CS (●) in linear relation with the factor of volumetric concentration (fc) and (**f**), progress of protein (○) and CS (●) retention with the increase in diavolume from DF process (D). Equation (3) was used to simulate the experimental data. Error bars are the confidence intervals (α = 0.05; n = 2).

Following the conditions summarized in Table 1, we performed a set of experiments to optimize the joint effect of *pH* and *T* on alcalase’s capacity to digest the skeleton of blue shark. The dependent variables were the concentration of CS obtained at the end of the hydrolysis and the index of the purity of the samples. In both responses, all the coefficients were statistically significant (t-Student test, α = 0.05) and quadratic terms for *T*^2^ and *pH*^2^ were negative (Table 2) conducting to convex shapes (Figure 2a,b). The theoretical surfaces predicted by the polynomial equations showed clear maximum points derivate from those convex forms. Correlation between experimental and predicted data for CS and *I_p_*, as R^2^ and R^2^_adj_, notably had values of 0.882–0.893 and 0.798–0.816, respectively. Ratios *F*1 and *F*2 from F-Fisher test validated the robustness of the equations (Table 2).

The search of the *T* and *pH* values that maximize the purity and the amount of CS recovery was made by numerical derivation: 56.1 °C/pH 8.42 for CS and 59.6 °C/pH 8.27 for *I_p_*. The average value of both conditions (57.9 °C/pH 8.35) was selected for the next step of the selective precipitation of the glycosaminoglycans. The purity of the sample reached in these levels was around 80% (presence of GAGs in relation to proteins) and the concentration of CS of 4.6 g/L. These outcomes are in concordance with the optimal values found for the hydrolysis of blue shark heads [28] and fins of small-spotted catshark [27]. As expected, the concrete best conditions for alcalase were dependent on the type of fish substrate employed [22,33,34].

A larger hydrolysate of 4 L, produced in those optimal conditions (pH = 8.35, 57.9 °C, t = 4 h, agitation = 200 rpm, E/S ratio = 1%) and obtained after centrifuge separation of solid material (all bioapatites was recovered in this stage), was used to study the process of selective precipitation of CS by chemical reagents. The concentration ranges of alkaline-hydroalcoholic solutions applied for this purpose followed the values reported in previous reports [28,33,34]: 0.1–0.8 M NaOH and 0.3–1.4 v of EtOH (Table 1).

In Figure 2c,d, the predicted response surfaces for the alkaline-ethanolic treatment of the hydrolysates are depicted. Once again, the surface trends and the second order equations were similar: all coefficients were statistically significant, including linear, mixed (*N* × *E*) and quadratic terms, and the latter are always negative (Table 3). The correlation among experimental and simulated data was also remarkable R^2^ = 0.888–0.889 and R^2^_adj_ = 0.808–0.809, and the consistency of equations was confirmed after overcoming both ratios from *F*-Fisher tests. Optimal conditions for *I_p_* and CS responses were quite closed: 0.44–0.52 M NaOH and 1.05–1.09 v of EtOH. From these data, and using the same compromise option defined in the previous factorial experiments, the best global values are 0.48 M/1.07 v. The concentration of CS isolated under those conditions was around 4.9 g/L with a 93% purity. This chemical step improved the purity of CS by more than 10% in comparison with the CS liberated from enzyme hydrolysis.

In a similar way, CS from wastes of small-spotted catshark, chimera and blackmouth catshark were adequately recovered in the range of 0.45–0.65 M of alkalis and 1.0–1.4 v of ethanol, increasing in all cases the purity of the solutions generated in the previous hydrolysis [28,34].

For the purification by membranes, a high solution of impure CS was produced on the conditions of hydrolysis (pH = 8.35, 57.9 °C, t = 4 h, agitation = 200 rpm, E/S ratio = 1%) and chemical treatment (0.48 M of NaOH/1.07 v of EtOH) previously optimized. This solution of CS, with a 93–94% of purity, was processed by ultrafiltration membrane (UF-30 kDa cut-off) with the aim to improve this value, removing peptides and short proteins throughout the membrane pores. Experimental data of concentration factors for protein retentates display null correlation with the simulated values, revealing low protein retention (Figure 2e). Conversely, the agreement between experimental and predicted data for CS was accurate, describing a complete retention of the biopolymer. Both tendencies were confirmed in the next diafiltration (DF) experiments where CS was fully retained and protein almost completely filtrated after more than 6 diavolumes. The flow of permeates, under constant transmembrane pressure of 1 bar, was 279 ± 23 mL/min. Experimental data of DF for CS and protein were adequately modelled by exponential Equation (3) with R^2^ > 0.985 in both cases. The values of the specific retention (*s*) from that mathematical model were of 0.974 ± 0.123 for CS and 0.571 ± 0.103 for protein, indicating high and low retention of CS and protein, respectively.

Retentates dried in the oven showed a purity in CS of 96.9 ± 0.5% and very low concentration of salts (<0.5% *w/w*), indicating an almost exhaustive desalination of samples by the DF step. The yield of final CS recovered from the central skeleton of the blue shark was 2.8 ± 0.1% (*w*/*w* of wet cartilage). This value was lower than those found for other chondrichthyes species (catshark, chimera) and fish cartilaginous tissues (heads, fins) [27,33], but the large amount of this residue deposited in shark processing industries makes the production of this biopolymer equally interesting. In sharks, the part of the body richer in CS is the head, achieving up to 12% (*w/w* of dry cartilage) in the blue shark [28].

In terms of purification strategies, we have developed a set of steps based on enzymatic, chemical and membrane operations working at optimal, reproducible and efficient conditions. In previous reports similar procedures have also demonstrated viability in the recovery of CS from other fish by-products [35,36]. The protocols for the isolation of GAGs in mammalian are mainly carried out using reagents such as cetylpyridinium chloride or cetyltrimethylammonium bromide [37,38] combined with chromatographic separations [38,39]. Both proposals are expensive and time-consuming in comparison with the present scheme, which furthermore is more easily reproducible at the pilot plant [40,41,42]. We must take into consideration that fishing activities is not a sector accustomed to making sustainable use of its waste and discards to obtain products with high added value. In this way, any proposal aimed at this end must be economically viable, using easily controllable and scalable processes and equipment.

On the other hand, the quality of CS isolated from the skeleton makes it compatible to serve as an ingredient for the formulation of different nutraceutical and pharmaceutical compounds. CS is widely prescribed for osteoarthritis diseases [43], employing biopolymers that must present high standard purities [44]. In a recent report, several pharmaceutical-grade CS were analysed, obtaining levels of purity in the range of 95–99% [45], being able to include our CS (96.9%) in this category. In the case of nutraceuticals, the purity of CS needed is lower and never higher than 85% [46].

### 3.2. Chemical Characterization of CS

**Figure 3:**
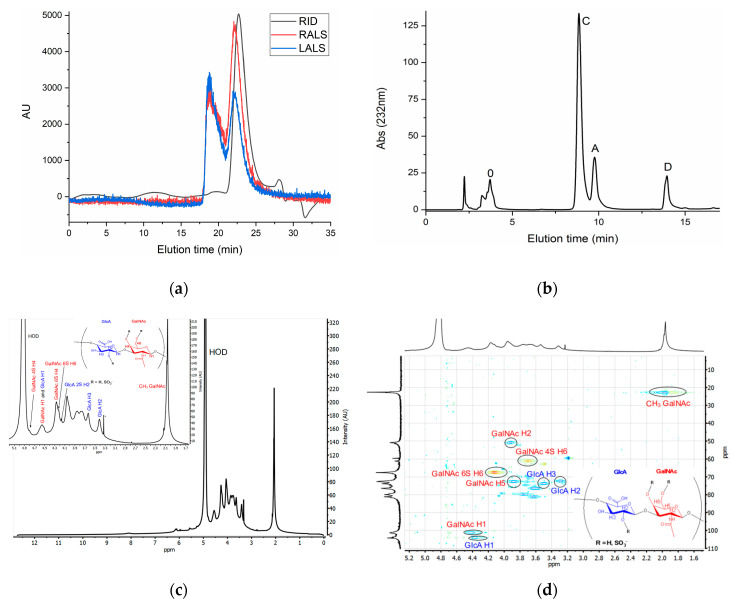
(**a**)—Gel Permeation Chromatography (GPC) eluograms of CS extracted from *P*. glauca. Red line: right angle light scattering signal (RALS); Blue line: low angle light scattering signal (RALS); Black line: refractive index (RID) signal; AU: arbitrary units. (**b**)—SAX-HPLC chromatogram of CS from from *P*. glauca after enzymatic digestion with chondroitinase ABC. 0: ΔUA-GalNAc; A: ΔUA-GalNAc(4S); C: ΔUA-GalNAc(6S); D: ΔUA(2S)-GalNAc(6S). Full ^1^H NMR with expansion of region with CS signals (**c**) and HSQC (**d**) spectra of CS extracted from *P*. glauca in D_2_O recorded at 10 °C.

Absolute molecular weight estimations of skeleton CS by GPC result in a value of 58 kDa (*M*_n_, number average molecular weight) and PDI (polydispersity index) of 1.16 (Table 3). This figure is comparable to values previously reported for this shark species, with CS isolated from the jaw at 67 kDa [8], from the skin at 70 kDa [47], and from whole heads at 60 kDa [48]. In the GPC eluogram, the CS peak shows at 22.72 min (Figure 3a). However, another peak appears at 19.78 min in the light scattering detector but is not visible in the refractive index detector, indicating large molecular weight species in minute amounts. Such species possibly correspond to aggregation of CS molecules, as a similar behavior has been previously reported in CS [8,34], as well as in other glycosaminoglycans [49] and chitosan [50,51].

NMR spectra allows unequivocal identification of CS (Figure 3c,d), with characteristic signals at 2.06 ppm corresponding to acetyl protons in GalNAc, and typical overlapping of ring protons between 3–5 ppm. Assignment of some signals in this region is possible thanks to the HSQC spectrum (Figure 3d), such as those corresponding to the overlapping anomeric carbons of GalNAc and GlcA at 4.55 ppm and sulfation in carbon 6 of GalNAc, typical of shark CS [52,53]. Contamination with other glycosaminoglycans common in CS samples seems unlikely due to a lack of typical signals of keratan sulfate (H1 of GlcNAc at 4.71 ppm) or dermatan sulfate (H1 and H2 of IdoA at 4.87 and 3.52 ppm respectively) [54]. However, several unidentified signals appear between 5 and 6.2 ppm, as well as a signal at 8.1 ppm possibly corresponding to aromatic amino acids. In all cases, its low intensity suggests minute contamination.

Disaccharide analysis by SAX-HPLC (Figure 3b) reveals that the majority of units are monosulfated in positions 6 and 4 of GalNAc (64.7% and 16% respectively), followed by disulfated D-units (GlcA 2S-GalNAc 6S) at 12.2% and non-sulfated at 6.3%. Other disulfated units are present only in minute amounts (Table 3). These results are in agreement with the composition of other CS samples from *P. glauca* previously reported (10–13% GlcA-GalNAc 4S, 64–68% GlcA-GalNAc 6S, 7%–16% GlcA-GalNAc 0S, 9–11% GlcA 2S-GalNAc 6S) [8,48].

### 3.3. Bioapatite Characterization

**Figure 4:**
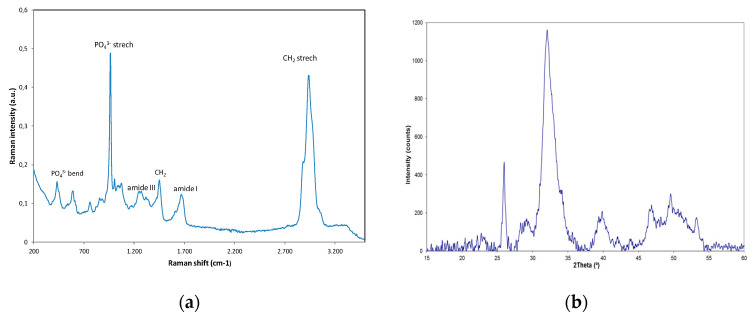
Raman spectrum (**a**) and XRD pattern (**b**) of crude bioapatites extracted from the central skeleton of blue shark (*P. glauca*).

The analysis of crude bioapatite through FT-Raman spectroscopy (Figure 4a) shows the presence of peaks associated with mineral phases of calcium phosphate, with an intense and narrow band at 960 cm^−1^ corresponding to PO_4_^3−^ symmetric stretching bonds, as well as the peaks at 1079 and 1048 cm^−1^ for PO_4_^3−^ asymmetric stretching, 428 and 445 cm^−1^ of symmetric bending modes, and 578, 582 and 606 cm^−1^ assigned to asymmetric bending of PO_4_^3−^ bonds [55]. Raman spectrum also reveal the presence of vibrational modes associated with organic compounds in the range 1200–3050 cm^−1^, being the peaks located at 1270, 1449 and 1667 cm^−1^ attributed to amide III, CH_2_ groups and amide I, respectively [56]. The broad band at 2850–3050 cm^−1^ is associated to C–H stretching modes of amide III in proteins and lipids [55].

The presence of calcium phosphate compounds was confirmed with XRD analysis (Figure 4b). The diffraction pattern corresponds to a mineralized tissue which has a percentage of inorganic components consisting of salts embedded in an organic matrix. The pattern shows the main peaks located at positions 25.94°, 29.24°, 32.06°, 34.18°, 39.92°, 49.59°, and 53.18°, which are characteristic of hydroxyapatite [Ca_5_(PO_4_)_3_(OH)], corresponding, respectively, to (0,0,2), (1,2,0), (2,1,1), (2,0,2), (3,1,0), (2,1,3), and (0,0,4) diffraction planes [55]. Broad reflection bands characteristic of mineralized tissues exhibit poor resolution which hinders the accurate identification and quantification of apatite and non-apatite crystalline phases of the mineral compounds.

## 4. Conclusions

The optimal recovery of CS and bioapatites from skeleton wastes of blue shark was studied here. The three main processes involved (enzyme hydrolysis, chemical treatment and membrane purification) were optimized using response surface methodology and non-linear function modelling. Thus, the isolation of CS and bioapatite was maximized by alcalase hydrolysis of skeleton at 1% (*v/w*) of enzyme, pH 8.35, 58 °C for 4 h. Then, increasing of CS purity from hydrolysates were defined by alkaline-hydroalcoholic saline treatment with 0.48 M NaOH, 1.07 v EtOH and NaCl 2.5 g/L. Combining UF and DF operation modes, employing a membrane of 30 kDa cut-off weight, CS was purified up to *I_p_* = 97% after adding 6–7 diavolumes of distilled water. The size of this biopolymer was 58 kDa as the number average molecular weight (*M*_n_) and the sulfation pattern indicated the predominance of dissacharides sulphated in C6 (4S/6S ratio of 0.25) with the presence of GlcA 2S-GalNAc 6S (12%) and 6% of unsulfated disaccharides. The analysis of crude bioapatite by FT-Raman and XRD techniques confirms the presence of hydroxyapatite compounds [Ca_5_(PO_4_)_3_(OH)] embedded in an organic matrix. The central skeleton of blue shark is a promising new marine source for the extraction of high value ceramic biomaterials. Further experiments must be performed to validate the capacity of these CS and bioapatites to be included in scaffolds, composites or nanodevices for the regeneration of cartilaginous and bone tissues.

## Figures and Tables

**Table 1:** Experimental domains and codification of the independent variables in the factorial rotatable designs performed to optimize the enzyme hydrolysis of blue shark skeletons and the chemical treatments of the hydrolysates using alkaline-hydroalcoholic solutions.

	Real Values			
Coded Values	Enzyme Hydrolysis		NaOH-EtOH Treatment	
	pH	*T* (°C)	NaOH (M)	Ethanol (*v*)
−1.41	6.0	30.0	0.10	0.30
−1	6.6	37.3	0.20	0.46
0	8.0	55.0	0.45	0.85
+1	9.4	72.7	0.70	1.24
+1.41	10.0	80.0	0.80	1.40

Codification: *V*_c_ = (*V*_n_ − *V*_0_)/∆*V*_n_; decodification: *V*_n_ = *V*_0_ + (∆*V*_n_ × *V*_c_); *V*_c_ = codified value of the variable; ∆*V*_n_ = increment of *V*_n_ per unit of *V*_c_; *V*_n_ = natural value of the variable to codify; *V*_0_ = natural value in the center of the domain.

**Table 2:** Polynomial equations describing the effect of *T* and *pH* on alcalase skeleton hydrolysis (CS isolation and *I_p_*-index) and characterising the effect of NaOH (*N*) and EtOH (*E*) on the precipitation of previous hydrolysates. The coefficient of determination and adjusted determination (*R*^2^ and *R*^2^*_adj_*), F-values and optimal conditions of the independent variables are also shown. S: significant.

	Skeletons Hydrolysis			Chemical Treatment	
Parameters	*CS*	*I_p_*	Parameters	*CS*	*I_p_*
**b_0_**	4.44 ± 0.25	79.77 ± 2.57	**b_0_**	4.51 ± 0.20	89.08 ± 2.46
**b_1_ (*T*)**	0.34 ± 0.20	4.14 ± 2.03	**b_1_ (*N*)**	0.28 ± 0.22	−5.86 ± 1.95
**b_2_ (*pH*)**	0.61 ± 0.20	6.35 ± 2.03	**b_2_ (*E*)**	1.16 ± 0.22	17.90 ± 1.95
**b_12_ (*T*** **× *pH*** **)**	−0.55 ± 0.28	−6.35 ± 1.46	**b_12_ (*N*** **× *E*** **)**	0.64 ± 0.32	9.13 ± 2.75
**b_11_ (*T*^2^)**	−1.44 ± 0.21	−5.57 ± 2.87	**b_11_ (*N*^2^)**	−1.13 ± 0.24	−16.31 ± 2.09
**b_22_ (*pH*^2^)**	−0.98 ± 0.21	−12.19 ± 2.87	**b_22_ (*E*^2^)**	−1.09 ± 0.24	−16.86 ± 2.09
***R*** **^2^** ***_adj_***	0.798	0.816	***R*** **^2^** ***_adj_***	0.809	0.808
***F1***	10.49 $[F_{7}^{5}=3.97]\Rightarrow S$	11.67 $[F_{7}^{5}=3.97]\Rightarrow S$	***F*** **1**	11.17 $[F_{7}^{5}=3.97]\Rightarrow S$	11.11 $[F_{7}^{5}=3.97]\Rightarrow S$
***F*** **2**	0.704 $[F_{5}^{8}=4.82]\Rightarrow S$	0.694 $[F_{5}^{8}=4.82]\Rightarrow S$	***F*** **2**	0.699 $[F_{5}^{8}=4.82]\Rightarrow S$	0.702 $[F_{5}^{8}=4.82]\Rightarrow S$
	***CS***	***I_p_***		***CS***	***I_p_***
***T_opt_*** **(°C)**	56.1	59.6	***NaOH_opt_*** **(M)**	0.52	0.44
***pH_opt_***	8.42	8.27	***EtOH_opt_*** **(v)**	1.09	1.05
***Y_max_***	4.54 g/L	80.9%	***Y_max_***	4.91 g/L	93.9%

**Table 3:** Molecular weight and disaccharide composition of CS from *P. glauca*. *M*_n_: number average molecular weight, PDI: polydispersity index (*M*_w_/*M*_n_); Disaccharide composition expressed as mean % ± standard deviation (n = 2).

Parameters and Dissacharides	Values
Mn	58 kDa
PDI	1.16
CS-A (GlcA-GalNAc 4S)^2^	15.97 ± 1.83
CS-C (GlcA-GalNAc 6S)^2^	64.72 ± 1.22
CS-0 (GlcA-GalNAc 0S)^2^	6.33 ± 1.86
CS-D (GlcA 2S-GalNAc 6S)^2^	12.23 ± 0.90
CS-E (GlcA-GalNAc 4,6S)^2^	0.38 ± 0.09
CS-B (GlcA 2S-GalNAc 4S)^2^	0.36 ± 0.27

## References

[1] Blanco M., Sotelo C.G., Chapela M.J., Pérez-Martín R.I. (2007). Towards sustainable and efficient use of fishery resources: Present and future trends. Trends Food Sci. Technol..

[2] Vázquez J.A., Durán A.I., Menduíña A., Nogueira M., Fraguas J., Mirón J., Valcárcel J. (2019). Tailor-made process to recover high added value compounds from fishery by-products. Green Extraction and Valorization of By-Products from Food Processing.

[3] Grosso C., Valentão P., Ferreres F., Andrade P.B. (2014). Bioactive marine drugs and marine biomaterials for brain diseases. Mar. Drugs.

[4] Venkatesan J., Kim S.-K., Kim S.-K. (2015). Marine biomaterials. Springer Handbook of Marine Biotechnology.

[5] Zhang C., Li X., Kim S.-K. (2012). Application of marine biomaterials for nutraceuticals and functional foods. Food Sci. Biotechol..

[6] De Iuliis G., Pulerà D. (2019). The Shark. Dissection of Vertebrates.

[7] López-Álvarez M., Pérez-Davila S., Rodríguez-Valencia C., González P., Serra J. (2016). The improved biological response of shark tooth bioapatites in a comparative in vitro study with synthetic and bovine bone grafts. Biomed. Mater..

[8] López-Álvarez M., González P., Serra J., Fraguas J., Valcarcel J., Vázquez J.A. (2020). Chondroitin sulfate and hydroxyapatite from *Prionace glauca* shark jaw: Physicochemical and structural characterization. Int. J. Biol. Macromol..

[9] Valcarcel J., Novoa-Carballal R., Pérez-Martín R.I., Reis R.L., Vázquez J.A. (2017). Glycosaminoglycans from marine sources as therapeutic agents. Biotechnol. Adv..

[10] Yamada S., Sugahara K. (2008). Potential therapeutic application of chondroitin sulfate/dermatan sulfate. Curr. Drug Discov. Technol..

[11] Celikkin N., Rinoldi C., Costantini M., Trombetta M., Rainer A., Święszkowski W. (2017). Naturally derived proteins and glycosaminoglycan scaffolds for tissue engineering applications. Mater. Sci. Eng. C.

[12] Lima M., Rudd T., Yates E. (2017). New applications of heparin and other glycosaminoglycans. Molecules.

[13] Pomin V.H. (2015). A dilemma in the glycosaminoglycan-based therapy: Synthetic or naturally unique molecules?. Med. Res. Rev..

[14] Murugan S., Sugahara K.N., Lee C.M., ten Dam G.B., van Kuppevelt T.H., Miyasaka M., Yamada S., Sugahara K. (2009). Involvement of chondroitin sulfate E in the liver tumor focal formation of murine osteosarcoma cells. Glycobiology.

[15] Pomin V.H. (2014). Holothurian fucosylated chondroitin sulfate. Mar. Drugs.

[16] Chen W.-C., Wei Y.-H., Chu I.M., Yao C.-L. (2013). Effect of chondroitin sulphate C on the in vitro and in vivo chondrogenesis of mesenchymal stem cells in crosslinked type II collagen scaffolds. J. Tissue Eng. Regen. Med..

[17] da Costa D.S., Reis R.L., Pashkuleva I. (2017). Sulfation of glycosaminoglycans and its implications in human health and disorders. Annu. Rev. Biomed. Eng..

[18] Nandini C.D., Mikami T., Ohta M., Itoh N., Akiyama-Nambu F., Sugahara K. (2004). Structural and functional characterization of oversulfated chondroitin sulfate/dermatan sulfate hybrid chains from the notochord of hagfish: Neuritogenic and binding activities for growth factors and neurotrophic factors. J. Biol. Chem..

[19] López-Senra E., Casal-Beiroa P., López-Álvarez M., Serra J., González P., Valcarcel J., Vázquez J.A., Burguera E.F., Blanco F.J., Magalhães J. (2020). Impact of prevalence ratios of chondroitin sulfate (CS)- 4 and -6 isomers derived from marine sources in cell proliferation and chondrogenic differentiation processes. Mar. Drugs.

[20] Antelo L.T., de Hijas-Liste G.M., Franco-Uría A., Alonso A.A., Pérez-Martín R.I. (2015). Optimisation of processing routes for a marine biorefinery. J. Clean. Prod..

[21] Seghetta M., Hou X., Bastianoni S., Bjerre A.-B., Thomsen M. (2016). Life cycle assessment of macroalgal biorefinery for the production of ethanol, proteins and fertilizers—A step towards a regenerative bioeconomy. J. Clean. Prod..

[22] Vázquez J.A., Ramos P., Valcarcel J., Antelo L.T., Novoa-Carballal R., Reis R.L., Pérez-Martín R.I. (2018). An integral and sustainable valorisation strategy of squid pen by-products. J. Clean. Prod..

[23] Vázquez J.A., Fraguas J., Mirón J., Valcárcel J., Pérez-Martín R.I., Antelo L.T. (2020). Valorisation of fish discards assisted by enzymatic hydrolysis and microbial bioconversion: Lab and pilot plant studies and preliminary sustainability evaluation. J. Clean. Prod..

[24] Vázquez J.A., Noriega D., Ramos P., Valcarcel J., Novoa-Carballal R., Pastrana L., Reis R.L., Pérez-Martín R.I. (2017). Optimization of high purity chitin and chitosan production from Illex argentinus pens by a combination of enzymatic and chemical processes. Carbohydr. Polym..

[25] Murado M.A., Montemayor M.I., Cabo M.L., Vázquez J.A., González M.P. (2012). Optimization of extraction and purification process of hyaluronic acid from fish eyeball. Food Bioprod. Process..

[26] Box G.E., Hunter J.S., Hunter W.G. (2005). Statistics for Experimenters: Design, Innovation, and Discovery.

[27] Blanco M., Fraguas J., Sotelo C.G., Pérez-Martín R.I., Vázquez J.A. (2015). Production of Chondroitin Sulphate from Head, Skeleton and Fins of Scyliorhinus canicula By-Products by Combination of Enzymatic, Chemical Precipitation and Ultrafiltration Methodologies. Mar. Drugs.

[28] Vázquez J.A., Blanco M., Fraguas J., Pastrana L., Pérez-Martín R. (2016). Optimisation of the extraction and purification of chondroitin sulphate from head by-products of Prionace glauca by environmental friendly processes. Food Chem..

[29] Amado I.R., Vázquez J.A., González M.P., Murado M.A. (2013). Production of antihypertensive and antioxidant activities by enzymatic hydrolysis of protein concentrates recovered by ultrafiltration from cuttlefish processing wastewaters. Biochem. Eng. J..

[30] Lowry O.H., Rosebrough N.J., Farr A.L., Randall R.J. (1951). Protein measurement with the Folin phenol reagent. J. Biol. Chem..

[31] van den Hoogen B.M., van Weeren P.R., Lopes-Cardozo M., van Golde L.M.G., Barneveld A., van de Lest C.H. (1998). A microtiter plate assay for the determination of uronic acids. Anal. Biochem..

[32] Murado M.A., Vázquez J.A., Montemayor M.I., Cabo M.L., González M.P. (2005). Two mathematical models for the correction of carbohydrate and protein interference in the determination of uronic acids by the *m*-hydroxydiphenyl method. Biotechnol. Appl. Biochem..

[33] Vázquez J.A., Fraguas J., Novoa-Carballal R., Reis R.L., Pérez-Martín R.I., Valcarcel J. (2019). Optimal isolation and characterisation of chondroitin sulfate from rabbit fish (*Chimaera monstrosa*). Carbohydr. Polym..

[34] Vázquez J.A., Fraguas J., Novoa-Carvallal R., Reis R., Antelo L.T., Pérez-Martín R.I., Valcarcel J. (2018). Isolation and chemical characterization of chondroitin sulfate from cartilage by-products of Blackmouth Catshark (*Galeus melastomus*). Mar. Drugs.

[35] Vázquez J.A., Rodríguez-Amado I., Montemayor M.I., Fraguas J., González M.D.P., Murado M.A. (2013). Chondroitin sulfate, hyaluronic acid and chitin/chitosan production using marine waste sources: Characteristics, applications and eco-friendly processes: A review. Mar. Drugs.

[36] Murado M.A., Fraguas J., Montemayor M.I., Vázquez J.A., González P. (2010). Preparation of highly purified chondroitin sulphate from skate (*Raja clavata*) cartilage by-products. Process optimization including a new procedure of alkaline hydroalcoholic hydrolysis. Biochem. Eng. J..

[37] Higashi K., Takeuchi Y., Mukuno A., Tomitori H., Miya M., Linhardt R.J., Toida T. (2015). Composition of glycosaminoglycans in elasmobranchs including several deep-sea sharks: Identification of chondroitin/dermatan sulfate from the dried fins of *Isurus oxyrinchus* and *Prionace glauca*. PLoS ONE.

[38] Souza A.R.C., Kozlowski E.O., Cerqueira V.R., Castelo-Branco M.T.L., Costa M.L., Pavão M.S.G. (2007). Chondroitin sulfate and keratan sulfate are the major glycosaminoglycans present in the adult zebrafish *Danio rerio* (Chordata-Cyprinidae). Glycoconj. J..

[39] Xie J., Ye H., Luo X. (2014). An efficient preparation of chondroitin sulfate and collagen peptides from shark cartilage. Int. Food Res. J..

[40] Arima K., Fujita H., Toita R., Imazu-Okada A., Tsutsumishita-Nakai N., Takeda N., Nakao Y., Wang H., Kawano M., Matsushita K. (2013). Amounts and compositional analysis of glycosaminoglycans in the tissue of fish. Carbohydr. Res..

[41] Maccari F., Galeotti F., Volpi N. (2015). Isolation and structural characterization of chondroitin sulfate from bony fishes. Carbohydr. Polym..

[42] Takeda N., Horai S., Tamura J.-I. (2016). Facile analysis of contents and compositions of the chondroitin sulfate/dermatan sulfate hybrid chain in shark and ray tissues. Carbohydr. Res..

[43] Reginster J.-Y., Cooper C., Hochberg M., Pelletier J.-P., Rizzoli R., Kanis J., Abadie E., Maheu E., Brandi M.L., Devogelaer J.P. (2015). Comments on the discordant recommendations for the use of symptomatic slow-acting drugs in knee osteoarthritis. Curr. Med. Res. Opin..

[44] Volpi N. (2009). Quality of different chondroitin sulfate preparations in relation to their therapeutic activity. J. Pharm. Pharmacol..

[45] da Cunha A.L., de Oliveira L.G., Maia L.F., de Oliveira L.F.C., Michelacci Y.M., de Aguiar J.A.K. (2015). Pharmaceutical grade chondroitin sulfate: Structural analysis and identification of contaminants in different commercial preparations. Carbohydr. Polym..

[46] Martel-Pelletier J., Farran A., Montell E., Vergés J., Pelletier J.-P. (2015). Discrepancies in composition and biological effects of different formulations of chondroitin sulfate. Molecules.

[47] Nandini C.D., Itoh N., Sugahara K. (2005). Novel 70-kDa Chondroitin Sulfate/Dermatan Sulfate Hybrid Chains with a Unique Heterogenous Sulfation Pattern from Shark Skin, Which Exhibit Neuritogenic Activity and Binding Activities for Growth Factors and Neurotrophic Factors. J. Biol. Chem..

[48] Novoa-Carballal R., Pérez-Martín R.I., Blanco M., Sotelo C.G., Fassini D., Nunes C., Coimbra M.A., Silva T.H., Reis R.L., Vázquez J.A. (2017). By-products of *Scyliorhinus canicula*, *Prionace glauca* and *Raja clavata*: A valuable source of predominantly 6S sulfated chondroitin sulfate. Carbohydr. Polym..

[49] Bertini S., Bisio A., Torri G., Bensi D., Terbojevich M. (2005). Molecular weight determination of heparin and dermatan sulfate by size exclusion chromatography with a triple detector array. Biomacromolecules.

[50] Lamarque G., Lucas J.-M., Viton C., Domard A. (2005). Physicochemical behavior of homogeneous series of acetylated chitosans in aqueous solution: Role of various structural parameters. Biomacromolecules.

[51] Ottøy M.H., Vårum K.M., Christensen B.E., Anthonsen M.W., Smidsrød O. (1996). Preparative and analytical size-exclusion chromatography of chitosans. Carbohydr. Polym..

[52] López-Álvarez M., López-Senra E., Valcárcel J., Vázquez J.A., Serra J., González P. (2019). Quantitative evaluation of sulfation position prevalence in chondroitin sulphate by Raman spectroscopy. J. Raman Spectrosc..

[53] Krylov V.B., Grachev A.A., Ustyuzhanina N.E., Ushakova N.A., Preobrazhenskaya M.E., Kozlova N.I., Portsel M.N., Konovalova I.N., Novikov V.Y., Siebert H.-C. (2011). Preliminary structural characterization, anti-inflammatory and anticoagulant activities of chondroitin sulfates from marine fish cartilage. Russ. Chem. Bull..

[54] Pomin V.H. (2013). NMR chemical shifts in structural biology of glycosaminoglycans. Anal. Chem..

[55] Aguiar H., Chiussi S., López-Álvarez M., González P., Serra J. (2018). Structural characterization of bioceramics and mineralized tissues based on Raman and XRD techniques. Ceram. Int..

[56] Valdés R., Stefanov S., Chiussi S., López-Alvarez M., González P. (2014). Pilot research on the evaluation and detection of head and neck squamous cell carcinoma by Raman spectroscopy. J. Raman Spectrosc..

